# Can Whole-Body Cryotherapy with Subsequent Kinesiotherapy Procedures in Closed Type Cryogenic Chamber Improve BASDAI, BASFI, and Some Spine Mobility Parameters and Decrease Pain Intensity in Patients with Ankylosing Spondylitis?

**DOI:** 10.1155/2015/404259

**Published:** 2015-07-26

**Authors:** Agata Stanek, Armand Cholewka, Jolanta Gadula, Zofia Drzazga, Aleksander Sieron, Karolina Sieron-Stoltny

**Affiliations:** ^1^School of Medicine with the Division of Dentistry in Zabrze, Department and Clinic of Internal Diseases, Angiology and Physical Medicine in Bytom, Medical University of Silesia, Batorego Street 15, 41-902 Bytom, Poland; ^2^Department of Medical Physics, Chelkowski Institute of Physics, University of Silesia, 4 Uniwersytecka Street, 40-007 Katowice, Poland; ^3^Department of Whole-Body Rehabilitation, Health Resort Ustron, 1 Sanatoryjna Street, 43-450 Ustron, Poland; ^4^School of Health Sciences in Katowice, Department of Physical Medicine, Chair of Physiotherapy, Medical University of Silesia, Medyków Street 12, 40-752 Katowice, Poland

## Abstract

The present study investigated whether whole-body cryotherapy (WBC) procedures could potentially have more beneficial effects on index of BASDAI and BASFI, pain intensity, and spine mobility parameters: Ott test, modified Schober test, chest expansion in ankylosing spondylitis (AS) patients, than kinesiotherapy procedures used separately. AS patients were exposed to a cycle of WBC procedures lasting 3 minutes a day, with a subsequent 60 minutes of kinesiotherapy or 60 minutes of kinesiotherapy only, for 10 consecutive days excluding weekend. After the completion of the cycle of WBC procedures with subsequent kinesiotherapy in the AS patients, BASDAI index decreased about 40% in comparison with the input value, whereas in the group of patients who received only kinesiotherapy it decreased only about 15% in comparison with the input value. After the completion of the treatment in the WBC group, BASFI index decreased about 30% in comparison with the input value, whereas in the kinesiotherapy group it only decreased about 16% in comparison with the input value. The important conclusion was that, in WBC group with subsequent kinesiotherapy, we observed on average about twice better results than in the group treated only by kinesiotherapy.

## 1. Introduction

Ankylosing spondylitis (AS) is a chronic, usually progressive inflammatory rheumatic disease affecting primarily the axial skeleton and sacroiliac joints. It usually begins in the second or third decade of life and tends to occur more often in males with a male to female ratio of roughly 2 to 1. The overall prevalence is between 0.1% and 1.4%. Chronic spinal inflammation can develop a complete fusion of the vertebrae, a process called ankylosis, which causes total loss of mobility of the spine. In addition, AS may affect peripheral joints, the skin, eyes, bowel, or lungs [1].

The main symptoms of the disease are pain and stiffness in the low back, upper buttock area, neck, and the remaining regions of the spine, which may lead to structural and functional impairments [2].

Although over the last years a revolution in the treatment of AS has taken place, in terms of improved understanding of disease pathophysiology, still in the 21st century, physiotherapy plays very important role and is recommended as a cornerstone in the management of AS, together with medication [3–5].

The primary goals of physiotherapy of the AS patient are to improve mobility and strength, prevent or decrease spinal deformity, improve chest expansion, reduce pain, and improve one's overall function and quality of life [6].

Recent studies have shown that a combination of pharmacological treatment and physical therapy gave synergetic effects and produced positive benefits on pain, function, and health-related quality of life in the AS patients [7–9].

One of the most efficient methods of physical medicine used in the treatment of many diseases of the locomotor system is cryotherapy using extremely low temperatures (below −100°C) applied for a short time (2-3 minutes) to stimulate physiological reactions of the human organism, in order to make more effective pharmacological treatment and kinesiotherapy [10]. Cryotherapy can be used as local cryotherapy and whole-body cryotherapy. The action of cryogenic temperatures causes in human organism several favorable and physiological reactions such as analgesic effect, neuromuscular effect, anti-inflammatory and antioedematous effect, and circulatory effect [11, 12].

Cryogenic temperatures applied for whole-body apart from aforementioned effects have also significant influence on psyche and endocrine and immune system [13–15].

The studies show that WBC procedures in AS patients do not influence ejection fraction, late ventricular potentials, and QT dispersion in patients without significant pathology of circulatory system [16, 17].

In addition cryogenic temperatures applied for whole body of AS patients have beneficial influence on adaptive processes of vegetative nervous system [18], profile lipid [19] as well as antioxidant status [20, 21], decrease of inflammatory process [22], and improvement of rheological blood properties [23].

## 2. Materials and Methods

### 2.1. Participants

The study protocol has been reviewed and approved by the Bioethical Committee of the Medical University of Silesia in Katowice (permission no. NN-6501-93/I/07), and all analyzed patients were informed about trial and gave written consent for inclusion in the study. All clinical investigations have been conducted according to the principles expressed in the Declaration of Helsinki (1964).

The study involved 48 nonsmoking male patients with ankylosing spondylitis who were divided by the physician into two groups with allocation ratio 2 : 1: 32 patients exposed to whole-body cryotherapy procedures with subsequent kinesiotherapy (WBC group, mean age 46.03 ± 1.20 years) and 16 men exposed only to kinesiotherapy procedures (kinesiotherapy group, mean age 46.63 ± 1.50 years), with no significant difference in mean age and body mass index between them.

The enrolment to the study was performed in the group of succeeding male patients, with definite diagnosis of AS who did not suffer from any other diseases, had no associated pathologies, and whose attending physician did not apply disease modifying antirheumatic drugs (DMARDs), biologic agents, or steroids. The AS patients were treated with NSAIDs, whose doses were not altered in the research course. All patients included in the trial fulfilled the modified New York Criteria for definite diagnosis of AS, which serve as the basis for the ASAS/EULAR recommendations [24]. Final selection to the study included only patients HLA B27 positive, who exhibited II and III radiographic grade of sacroiliac joint disease, who attended consulting unit in Health Resort in the period of subsidence of acute clinical symptoms, in order to qualify for sanatorium treatment (physiotherapy). The demographic data of the study subjects are shown in Table 1.

Before the study each patient was examined by a physician to exclude any coexisting diseases as well as any contraindications for whole-body cryotherapy procedures. Prior to the study a resting electrocardiogram was performed on all patients, and before each session of cryotherapy the blood pressure was measured in all the patients.

#### 2.1.1. Whole-Body Cryotherapy and Kinesiotherapy Procedures

Depending on the group, the AS patients were exposed to a cycle of whole-body cryotherapy (WBC) procedures lasting 3 minutes a day, with a subsequent 60 minutes of kinesiotherapy, or 60 minutes of kinesiotherapy only, for 10 consecutive days excluding weekend.

The whole-body cryotherapy procedures were performed in a liquid nitrogen cooled cryogenic chamber (CR 2000) (produced by Creator, Poland), which consists of two compartments: the antechamber and the proper chamber connected by an internal door. In the trial the temperature in the antechamber was −60°C, whereas in the proper chamber it reached −120°C. After a 30-second adaptation process in the antechamber, the subjects were exposed to cryogenic temperatures for 3 minutes in the proper chamber. During the WBC procedure, all patients were dressed in swimsuits, cotton socks and gloves, and wooden shoes, whereas their mouths and noses were protected by surgical masks and their ears by ear-protectors. All jewellery, glasses, and contact lenses were removed before entry into the chamber. During the WBC procedure the subjects were walking round the chamber without touching each other.

Immediately after leaving the cryogenic chamber and changing clothes and shoes (for track-suits and trainers), the AS patients underwent 1-hour long kinesiotherapy. The program of kinesiotherapy was the same for all patients in both groups. Kinesiotherapy procedures included range-of-motion exercise of the spine and major joints (including the ankle, knee, hip, wrist, elbow, and shoulder). Chest expansion and breathing exercises were also included. Apart from range-of-motion exercise, the AS patients received strengthening exercise of the muscles of the major joints (including the ankle, knee, hip, wrist, elbow, shoulder, thoracolumbar spine, and cervical spine) as well as aerobic exercise (including cycling and fast walking).

All patients completed the study and no complications or side effects related to the WBC procedures were observed.

### 2.2. Assessments

The primary outcome measures were BASDAI and BASFI index. Secondary measures included pain intensity and chosen spine mobility parameters.

On the first day before the treatment cycle of WBC and/or kinesiotherapy and on the first day after treatment completion, the following parameters were estimated: the Bath Ankylosing Spondylitis Diseases Activity Index (BASDAI), the Bath Ankylosing Spondylitis Functional Index (BASFI), and pain intensity by means of 10-point visual analogue scale (VAS). Participants' spinal mobility was also examined through Ott test, modified Schober test, and chest expansion.

The BASDAI has six questions related to fatigue, back pain, peripheral pain, peripheral swelling, local tenderness, and morning stiffness (degree and length). Other than the item relating to morning stiffness, all questions are scored from 0 (none) to 10 (very severe) using a visual analogue scale (VAS). A sum score was calculated as mean of two morning stiffness items and the four remaining items [25].

The BASFI is the mean score of ten questions addressing functional limitations and the level of physical activity at home and work, assessed on VAS scales (0 = easy, 10 = impossible) [26].

Ott test measures the range of motion of the thoracic spine. Patient standing and measurement made 30 cm inferior to the C7 spinous process. The measurement was repeated with patient in full forward flexion. An increase of less than 2 cm suggests decreased thoracic spinal mobility [27].

Modified Schober test measures the range of motion of the lumbar spine. Patient standing and measurements made 10 cm above and 5 cm below the lumbosacral junction (iliac crest line). The measurement was repeated with patient in full forward flexion. An increase of less than 6 cm suggests decreased lumbar spinal mobility [27].

The subject's chest expansion was taken in standing position with the feet 5 cm apart and arms elevated. The upper limbs at the sides with the shoulder abducted the elbow in semiflexion, the wrist extended, and the thumb abducted with the web between the thumb and the first fingers placed on the level of the iliac crest. The chest expansion was taken as the change in circumference of the patient's chest at the level of the 4th intercostal space at the end of forced inspiration minus thoracic circumference at the end of forced expiration. Limitation of chest expansion is where the patient's measure recorded in centimeters is less than the average normal value by a minimum of 2.5 cm correcting for age and gender [28].

### 2.3. Statistical Analysis

Statistical analysis was undertaken using the statistical package of Statistica 10 Pl software. For each parameter the indicators of the descriptive statistics were determined (mean value and standard deviation SD). The normality of the data distribution was checked using Shapiro-Wilk's test, while the homogeneity of the variance was verified with the use of Levene's test. In order to compare the differences between the initial values of particular laboratory parameters and values after the end of a cycle of treatment procedures in both groups of subjects, a dependent sample Student's *t*-test when homogeneity of variances and normality of distribution have been fulfilled, otherwise Wilcoxon signed-rank test was used. On the other hand in the case of independent (unpaired) samples the *t*-test or Mann-Whitney *U* test has been used which was also dependent on homogeneity of variances and normality of distribution.

Differences at a significance level of *p* < 0.05 were considered as statistically significant.

## 3. Results 

**Figure 1:**
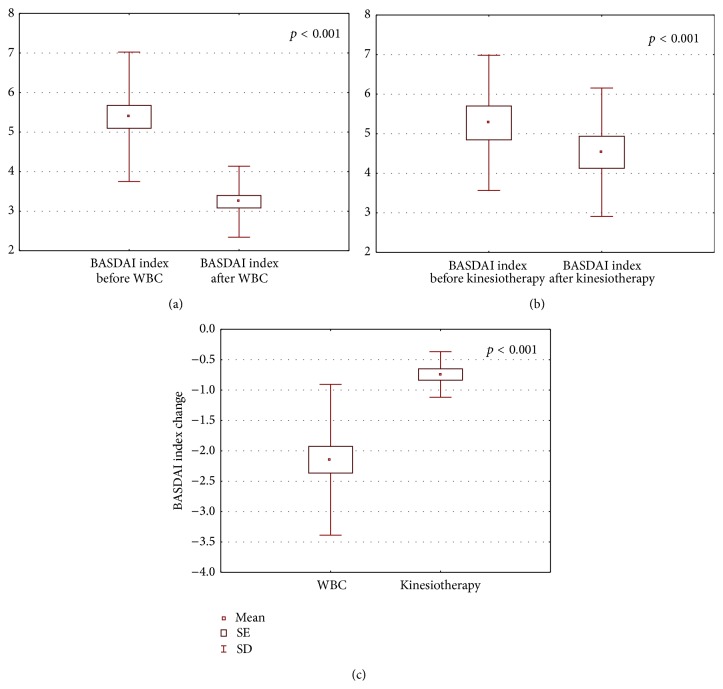
Comparison of BASDAI index before and after a cycle of whole-body cryotherapy (WBC) with subsequent kinesiotherapy procedures (a), before and after only a cycle of kinesiotherapy procedures (b), and BASDAI index change in whole-body cryotherapy and kinesiotherapy groups (c) obtained for patients suffering from AS.

**Figure 2:**
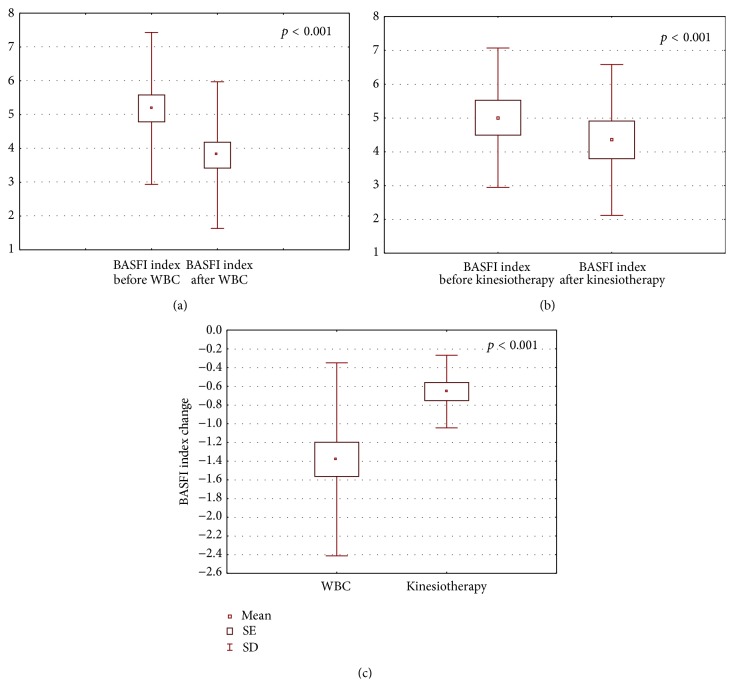
Comparison of BASFI index before and after a cycle of whole-body cryotherapy (WBC) with subsequent kinesiotherapy procedures (a), before and after only a cycle of kinesiotherapy procedures (b), and BASFI index change in whole-body cryotherapy and kinesiotherapy groups (c) obtained for patients suffering from AS.

**Figure 3:**
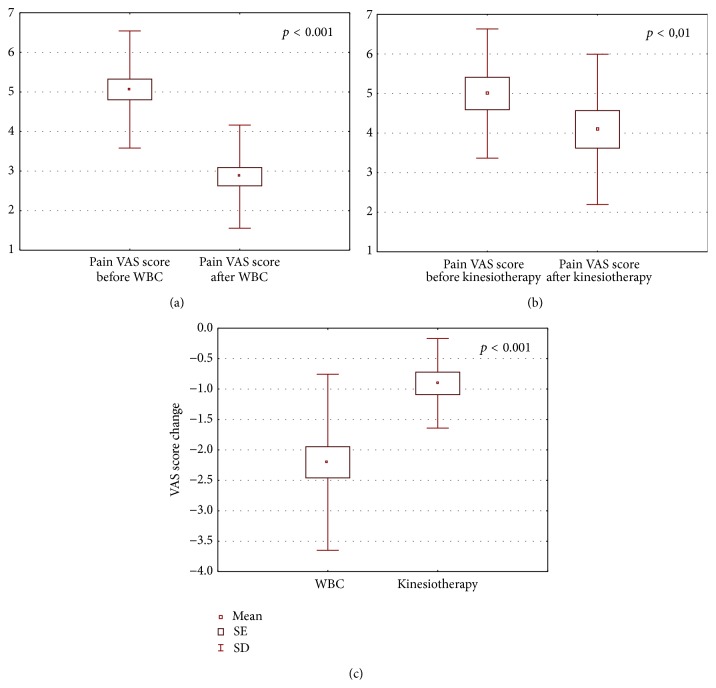
Comparison of pain VAS score before and after a cycle of whole-body cryotherapy (WBC) with subsequent kinesiotherapy procedures (a), before and after only a cycle of kinesiotherapy procedures (b), and VAS score change in whole-body cryotherapy and kinesiotherapy groups (c) obtained for patients suffering from AS.

**Figure 4:**
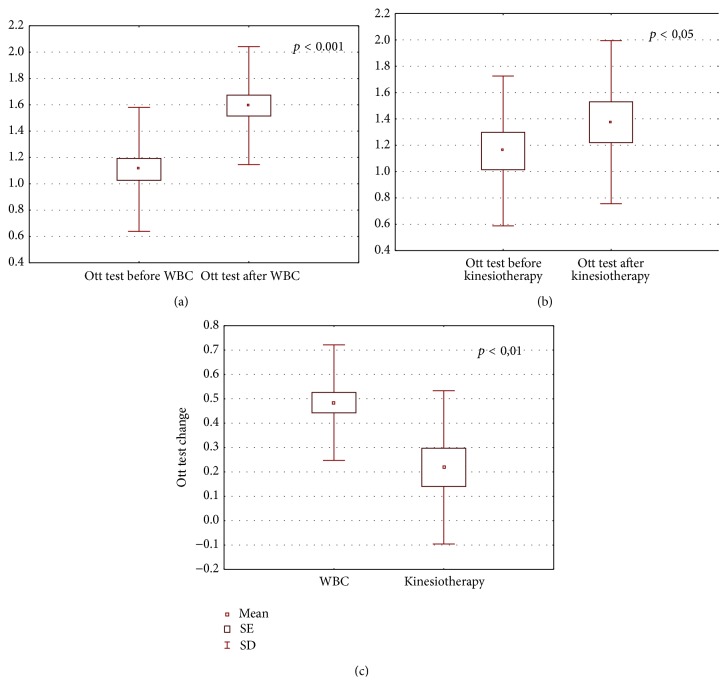
Comparison of Ott test (cm) before and after a cycle of whole-body cryotherapy (WBC) with subsequent kinesiotherapy procedures (a), before and after only a cycle of kinesiotherapy procedures (b), and Ott test change in whole-body cryotherapy and kinesiotherapy groups (c) obtained for patients suffering from AS.

**Figure 5:**
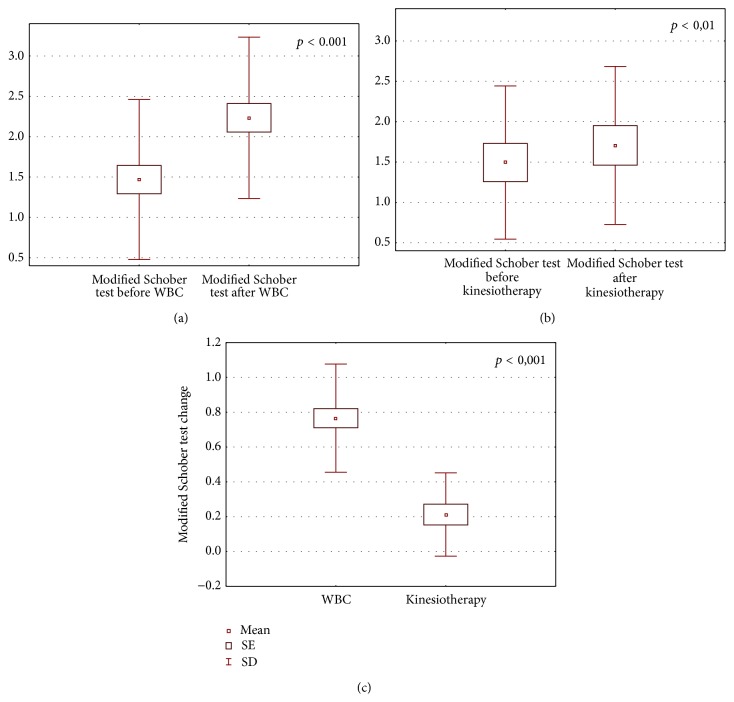
Comparison of modified Schober test (cm) before and after a cycle of whole-body cryotherapy (WBC) with subsequent kinesiotherapy procedures (a), before and after only a cycle of kinesiotherapy procedures (b), and Schober test change in whole-body cryotherapy and kinesiotherapy groups (c) obtained for patients suffering from AS.

**Figure 6:**
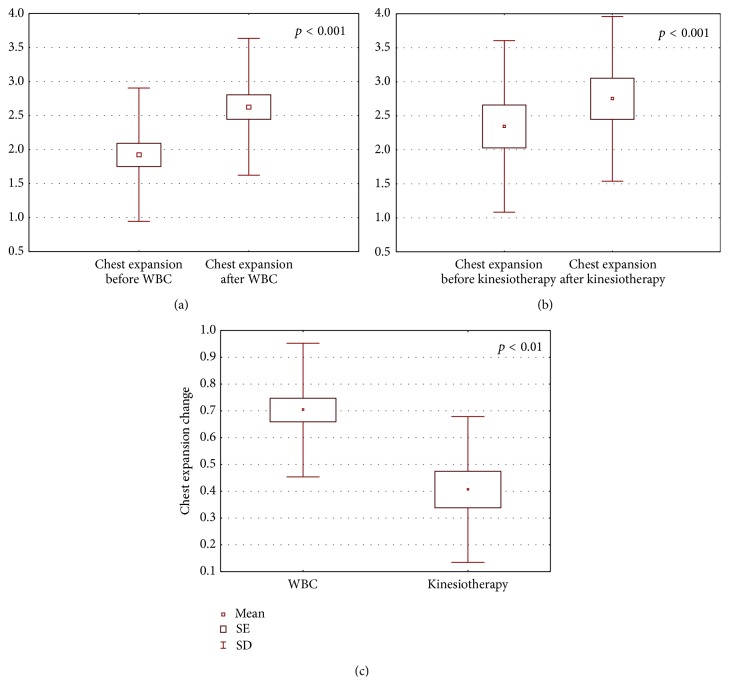
Comparison of chest expansion test (cm) before and after a cycle of whole-body cryotherapy with subsequent kinesiotherapy procedures (a), before and after only a cycle of kinesiotherapy procedures (b), and chest expansion test change in whole-body cryotherapy and kinesiotherapy groups (c) obtained for patients suffering from AS.

The obtained results are shown in Figures 1–6.

After the completion of the treatment in both studied groups, statistically significant improvement in the values of all examined parameters was noted.

After the completion of treatment in AS patients who underwent whole body cryotherapy procedures with subsequent kinesiotherapy (WBC group), a statistically significant decrease in BASDAI index (5.39 ± 1.64 and 3.24 ± 0.90 before and after therapy, resp., *p* < 0.001) (Figure 1(a)), BASFI index (5.18 ± 2.25 and 3.86 ± 2.1790 before and after therapy, resp., *p* < 0.001) (Figure 2(a)), and pain VAS score (5.34 ± 1.58 and 2.86 ± 1.28 before and after therapy, resp., *p* < 0.001) (Figure 3(a)) and a statistically significant increase in Ott test (1.11 ± 0.47 and 1.59 ± 0.45 before and after therapy, resp., *p* < 0.001) (Figure 4(a)), modified Schober test (1.47 ± 0.99 and 2.23 ± 1.00 before and after therapy, resp., *p* < 0.001) (Figure 5(a)), and chest expansion (1.92 ± 0.98 and 2.63 ± 1.00 before and after therapy, resp., *p* < 0.001) (Figure 6(a)) were obtained.

After the completion of treatment in AS patients who underwent only the kinesiotherapy procedures (kinesiotherapy group), a statistically significant decrease in BASDAI index (5.28 ± 1.71 and 4.53 ± 1.62 before and after therapy, resp., *p* < 0.001) (Figure 1(b)), BASFI index (5.01 ± 2.06 and 4.35 ± 2.23 before and after therapy, resp., *p* < 0.001) (Figure 2(b)), and pain VAS score (5.00 ± 1.63 and 4.09 ± 1.90 before and after therapy, resp., *p* < 0.01) (Figure 3(b)) and a statistically significant increase in Ott test (1.16 ± 0.57 and 1.38 ± 0.62 before and after therapy, resp., *p* < 0.05) (Figure 4(b)), modified Schober test (1.49 ± 0.95 and 1.71 ± 0.98 before and after therapy, resp., *p* < 0.01) (Figure 5(b)), and chest expansion (2.34 ± 1.26 and 2.75 ± 1.21 before and after therapy, resp., *p* < 0.001) (Figure 6(b)) were also observed.

However, in WBC group the examined parameter changes were significantly higher than in kinesiotherapy group: BASDAI change (−2.15 ± 1.24 in WBC group versus −0.74 ± 0.34 in kinesiotherapy group, *p* < 0.001) (Figure 1(c)), BASFI change (−1.38 ± 1.03 in WBC group versus −0.66 ± 0.39 in kinesiotherapy group, *p* < 0.001) (Figure 2(c)), pain VAS score change (−2.48 ± 1.79 in WBC group versus −0.91 ± 0.74 in kinesiotherapy group, *p* < 0.001) (Figure 3(c)), Ott test change (0.48 ± 0.24 in WBC group versus 0.22 ± 0.31 in kinesiotherapy group, *p* < 0.01) (Figure 4(c)), modified Schober test change (0.77 ± 0.31 in WBC group versus 0.21 ± 0.24 in kinesiotherapy group, *p* < 0.001) (Figure 5(c)), and chest expansion change (0.70 ± 0.25 in WBC group versus 0.41 ± 0.27 in kinesiotherapy group, *p* < 0.01) (Figure 6(c)).

## 4. Discussion

The available professional literature lacks studies assessing the influence of whole-body cryotherapy on AS patients, besides our own studies.

One of the most significant effects of cryogenic temperatures is the analgesic effect connected with influence of low temperatures on endocrine system (increased secretion of *β*-endorphins), nervous system (functional disconnection of sensory receptors and their connections with proprioreceptors, release of conductivity in slowly conductive fibers, and selecting impulses coming to nervous system, mechanism of “control gates”), and metabolic action (among others decreased concentrations of histamine and lactate in inflammatory changed tissues) [11, 29, 30].

In the study [31] AS patients assessed subjectively the WBC procedures effects by means of a questionnaire. The WBC procedures were performed in cryogenic chamber with cold retention. In cited study the significant improvement concerned mainly the reduction of the intensity and frequency of pain occurring as well as relaxation and improvement in the quality of falling asleep and sleep.

In our own study [32] we showed that in AS patients WBC procedures performed in cryogenic chamber with cold retention caused the reduction of pain intensity by 46% in comparison with the input value, whereas in the group of the patients who received only kinesiotherapy the pain intensity reduction amounted only to 18%.

In the presented study we observed similar results. The pain intensity decreased about 43% in the WBC group, whereas in kinesiotherapy group the decrease was only about 21%.

BASDAI index estimates the disease activity by evaluation of fatigue, axial pain, peripheral pain, stiffness, and enthesopathy in the AS patients. It is a quick and simple index and demonstrates a sensitivity to change within a short period of time as well as statistically significant (*p* < 0.001) reliability. The value of BASDAI index above 4 means that the disease is in an active period [25]. Before the trial in both examined groups the value of BASDAI index was above 4. After the completion of cycle of whole-body cryotherapy procedures with subsequent kinesiotherapy in the AS patients it decreased about 40% in comparison with the input value, whereas in the group of the patients who received only kinesiotherapy the BASDAI index decreased about 15% in comparison with the input value.

Moreover, after the completion of the treatment only in the WBC group its value was below 4 (inactive disease).

After pain and stiffness, one of the most important complaints of patients with AS is disability. The BASFI index determines the degree of functional limitation in the AS patients. The first 8 questions evaluate activities related to functional anatomical limitations due to the course of this inflammatory disease. The final 2 questions evaluate the patients' ability to cope with everyday life. Its sensitivity and reliability are similar to the BASDAI index [26]. As in the case of BASDAI index, the BASFI index value was above 4 in both studied groups. After completion of the treatment in the WBC group the value of BASFI index decreased about 30% in comparison with the input value, whereas in the kinesiotherapy group it only decreased about 16% in comparison with the input value. Moreover, similar to the BASDAI index, after the completion of the treatment only in the WBC group its value was below 4 (inactive disease).

In WBC group Ott test, modified Schober test, and chest expansion increased about 60%, 83%, and 53% in comparison to the input value, whereas in the kinesiotherapy group Ott test, modified Schober test, and chest expansion increased only about 26%, 16%, and 26% in comparison to the input value. We observed similar results in AS patients who underwent WBC procedures in the cryogenic chamber with cold retention [33].

Whole-body cryotherapy with subsequent kinesiotherapy effects in AS patients was explicitly better compared to the group of patients who received only kinesiotherapy. Beneficial effect of whole-body cryotherapy procedures on the value of the index BASDAI and BASFI and parameters of the spine mobility in the AS patients is mainly due to its anti-inflammatory, neuromuscular, and antioedematous as well as aforementioned analgesic action.

Anti-inflammatory effect of WBC procedures may arise both from the impact of cryogenic temperatures on secretion of mediators of inflammation and on the prooxidant-antioxidant balance and stabilization of lysosome membranes and subsequent inhibition of release of active enzymes from lysosomes.

WBC procedures cause the decrease in level of inflammatory state parameters; among others are erythrocyte sedimentation rate (ESR) value, serum concentration of C-reactive protein (CRP), fibrinogen, seromucoid, sICAM (soluble intercellular adhesion molecule), proinflammatory cytokines IL-1, IL-2, IL-6, and IL-8 levels, and increase in anti-inflammatory cytokine IL-10 level [22, 34–36].

The other mechanism of anti-inflammatory action of WBC procedures could be related to its beneficial influence on prooxidant-antioxidant balance. It is suggested that repeated exposures to cryogenic temperatures may cause adaptative changes in the form of an increase in antioxidant status and antioxidant enzyme activity, resulting in the formation of a prooxidant-antioxidant balance at a higher level, assisting in an anti-inflammatory effect and protecting tissues against an increased generation of reactive oxygen species and oxidative stress caused by training [37–39]. In our own study in AS patients after ten treatments we also observed the beneficial influence of WBC procedures on antioxidant status (increased activity of superoxide dismutase, level of total antioxidant status, and decreased level of malondialdehyde) [20].

The other mechanisms of anti-inflammatory action of whole-body cryotherapy may be linked to stabilization of lysosome membranes and subsequent inhibition of release of active enzymes from lysosomes [40]. It seems that this effect could be related to increased ACTH and cortisone blood concentrations, due to both WBC and physical training [41, 42].

The obtained results may be also connected with neuromuscular effect of cryotherapy which results in reduction of muscular tone (reduction of nerve transmission and reactivity of peripheral nerve endings) and increase of muscle strength [10, 29, 30].

The other effect of WBC which may have influence on achieved results is antioedematous action. This mechanism results in improvement in patency of lymphatic vessels draining intercellular space and increase in capillary filtration and acceleration of lymph flow [11].

Through these aforementioned mechanisms of WBC procedures, we can observe much better results compared to kinesiotherapy procedures used separately. WBC preceding kinesiotherapy allows intensification of the training and extension of the time of its duration several times [30]. Therefore, procedures of WBC and therapeutic exercises are altogether main components of the so-called cryorehabilitation [43–45].

In addition, observed calming and nervous tension reduction in AS patients after WBC treatment may increase the mobilization of patients to exercise and improve cooperation with the doctor and physiotherapeutist [31].

As authors' own experience and literature data show, WBC is well tolerated by patients, including children and the elderly. During the first days of procedure, there may occur slight aggravation of disease symptoms which is a generally promising prognosis. Patients admitted for the whole-body cryotherapy are instructed how to behave during the procedure. Special attention is paid to the way of breathing in the proper chamber during the procedure. Inhaling should be two times shorter than exhaling due to decompression of cooled air in lungs. Noncompliance with the recommendation may lead to serious breathing depression. Moreover, it is forbidden to touch other patients or rub own skin. In addition each time before WBC procedure patients should dry their skin with towel in order to remove sweat, as sweat drops turn into ice crystals in a cryochamber and it may lead to frostbite [11].

Our study has some limitations. First, the study did not provide long-term followup (e.g., 3 months), and thus we do not know how long the effect of whole-body cryotherapy with subsequent kinesiotherapy would be maintained after the end of study. Secondly, a cycle of whole-body cryotherapy (WBC) with a subsequent kinesiotherapy consisted of ten procedures only. A greater number of procedures (e.g., 20–30) might increase a treatment effect.

## 5. General Conclusion

In the present report, we demonstrated that whole-body cryotherapy procedures have beneficial influence on AS patients through decrease of BASDAI and BASFI index, pain intensity, and improvement of some spinal mobility parameters.

## Figures and Tables

**Table 1:** Demographic data of the study subjects.

Characteristic	WBC group with subsequent kinesiotherapy (*n* = 32)	Kinesiotherapy group (*n* = 16)	*p* value
Age, years, mean (SD)	46.03 ± 1.20	46.63 ± 1.50	0.095
Gender M/F	32/0	16/0	1.00
BMI, kg/m^2^, mean (SD)	24.1 ± 4.2	23.9 ± 5.8	0.564
Smoking (yes/no)	0/32	0/16	1.00
BASDAI index	5.39 ± 1,64	5.28 ± 1.71	0.767
BASFI index	5.18 ± 2.25	5.01 ± 2.06	0.965

Medication			
DMARD (yes/no)	0/32	0/16	1.00
Biological agents (yes/no)	0/32	0/16	1.00
NSAID (yes/no)	32/0	16/0	1.00

SD: standard deviation; BMI: body mass index; BASDAI: the Bath Ankylosing Spondylitis Diseases Activity Index; BASFI: the Bath Ankylosing Spondylitis Functional Index; NSAID: nonsteroidal anti-inflammatory drug, DMARD: disease-modifying antirheumatic drug.
